# Propofol exposure during early gestation impairs learning and memory in rat offspring by inhibiting the acetylation of histone

**DOI:** 10.1111/jcmm.13524

**Published:** 2018-02-20

**Authors:** Jiamei Lin, Shengqiang Wang, Yunlin Feng, Weihong Zhao, Weilu Zhao, Foquan Luo, Namin Feng

**Affiliations:** ^1^ Department of Anesthesiology the First Affiliated Hospital Nanchang University Nanchang China; ^2^ Department of Anesthesiology the Eastern Hospital of the First Affiliated Hospital Sun Yat‐sen University Guangzhou China

**Keywords:** histone deacetylase, learning and memory, pregnancy, propofol

## Abstract

Propofol is widely used in clinical practice, including non‐obstetric surgery in pregnant women. Previously, we found that propofol anaesthesia in maternal rats during the third trimester (E18) caused learning and memory impairment to the offspring rats, but how about the exposure during early pregnancy and the underlying mechanisms? Histone acetylation plays an important role in synaptic plasticity. In this study, propofol was administered to the pregnant rats in the early pregnancy (E7). The learning and memory function of the offspring were tested by Morris water maze (MWM) test on post‐natal day 30. Two hours before each MWM trial, histone deacetylase 2 (HDAC2) inhibitor, suberoylanilide hydroxamic acid (SAHA), Senegenin (SEN, traditional Chinese medicine), hippyragranin (HGN) antisense oligonucleotide (HGNA) or vehicle were given to the offspring. The protein levels of HDAC2, acetylated histone 3 (H3) and 4 (H4), cyclic adenosine monophosphate (cAMP) response element‐binding protein (CREB), N‐methyl‐D‐aspartate receptor (NMDAR) 2 subunit B (NR2B), HGN and synaptophysin in offspring's hippocampus were determined by Western blot or immunofluorescence test. It was discovered that infusion with propofol in maternal rats on E7 leads to impairment of learning and memory in offspring, increased the protein levels of HDAC2 and HGN, decreased the levels of acetylated H3 and H4 and phosphorylated CREB, NR2B and synaptophysin. HDAC2 inhibitor SAHA, Senegenin or HGN antisense oligonucleotide reversed all the changes. Thus, present results indicate exposure to propofol during the early gestation impairs offspring's learning and memory *via* inhibiting histone acetylation. SAHA, Senegenin and HGN antisense oligonucleotide might have therapeutic value for the adverse effect of propofol.

## 1 INTRODUCTION

Accumulating evidence indicates general anaesthetics exposure during pregnancy may cause neurotoxic effects and induce persistent cognitive dysfunction of offspring rats.^1^, ^2^, ^3^ Propofol is commonly used in pregnancy for non‐obstetric surgery. Xiong et al^4^ showed that anaesthesia with propofol on gestational day 18 (E18) associated with the up‐regulation of caspase‐3 and the loss of neurons, as well as associated with the down‐regulation synaptophysin expression in offspring rats’ hippocampus and caused persistent spatial learning impairment in offspring. Our previous study showed that propofol anaesthesia in the second trimester inhibits the cognitive function of the offspring that is related to down‐regulation of the protein levels of brain‐derived neurotrophic factor (BDNF) and synaptophysin in offspring hippocampus.^5^ Exposure to propofol for 5 hour caused death of neurons and oligodendrocytes in foetal and neonatal NHP brain.^6^ However, little attention was paid to the early stage of gestation, which is equivalent to the early pregnancy of human.^7^ It is reported that 0.75% to 2% gestational women have to experience non‐obstetric surgery due to various medical problems.^8^ This number is increasing with the development of laparoscopic technique, and the most common surgical procedure performed in the early pregnancy is laparoscopy.^9^ It is reported that about 28% of the non‐obstetric surgeries occurred in the first trimester.^10^ Our earlier studies demonstrated that propofol, ketamine, enflurane, isoflurane or sevoflurane anaesthesia in the early pregnancy inhibits the cognitive function, damages hippocampal neurons, reduces NR2B mRNA and increased HGN mRNA levels in offspring rats’ hippocampus,^11^, ^12^, ^13^, ^14^ but the underlying pathogenesis needs to be clarified.

Long‐term potentiation (LTP) is considered the cellular mechanism of memory formation and plays a role in synaptic plasticity.^15^ NR2B is an important positive regulator of learning and memory by promoting synaptic plasticity and LTP.^16^, ^17^ The balance between positive and negative learning and memory‐regulating genes and proteins is key to the formation, maintenance, as well as retrieval of memory. HGN is a negative regulating protein that highly expresses in hippocampus, acting suppression/clearance function in memory regulating.^18^ Inhibiting HGN by antisense oligonucleotide induces an increase in performance of Morris water maze and LTP. This indicates that HGN negatively regulates synaptic plasticity and LTP and plays negative regulating role in the formation and maintenance of memory.

Persistent changes in synapses, which based on appropriate gene transcription and subsequent protein synthesis, are the structural basis of learning and memory processes.^19^ Both compact chromatin structure and the accessibility of DNA to target genes can be modulated by chromatin remodelling, in particular, histone tail acetylation, thus to regulate their expression.^20^, ^21^ Histone acetylation regulates by acetyltransferases (HATs) and histone deacetylases (HDACs). HATs serve as transcriptional activators, whereas HDACs serve as transcriptional repressors. Increased HDAC activity had been linked to neurodegeneration. Growing evidence indicated that SAHA, which mainly targeting HDAC2, probably has therapeutic potentialities for the learning impairment caused by neurodegenerative diseases.^22^, ^23^, ^24^ Histone deacetylase inhibitors facilitated synaptic plasticity and memory by promoting the combination of CREB with CREB‐binding protein (CBP) domain, which subsequently activate CREB‐mediated transcription.^25^, ^26^, ^27^ Our early researches showed that anaesthesia during early gestation damaged the neurons and reduced the expression of NR2B in hippocampus, thus leading to learning and memory impairments in offspring rats.^11^, ^12^ In this study, we aim to investigate whether histone acetylation involves in the cognitive function impairment induced by propofol anaesthesia during early pregnancy.

## 2 MATERIALS AND METHODS

### 2.1 Drugs

All drugs were prepared just before use: propofol (Diprivan; AstraZeneca UK limited, Italy: jc393, 20 mL: 200 mg); 20% intralipid (2B6061; Baxter, Deerfield, IL, USA); SAHA (Selleck Chemicals LLC, Houston, TX, USA). HGN antisense was synthesized by Sangon Biotech (Shanghai, China) Co., Ltd. Senegenin (purity ≥ 98%) was purchased from Nanjing SenBeiJia Biological Technology Co., Ltd. (Jiangsu province, China).

Anti‐β‐actin and anti‐rabbit IgG secondary antibody were obtained from Cell Signaling Technology (Cell Signaling Tech, MA, USA). Anti‐CREB (Phospho S133), anti‐NMDAR2B, anti‐HDAC2, antisynaptophysin, anti‐Ac‐H4K12 and anti‐Ac‐H3K14 antibodies were purchased from Abcam (Abcam, Cambridge, MA, USA). Anti‐HGN antibody was synthesized by Kitgen Bio‐tech Co., Ltd.(Zhejiang province, China).

### 2.2 Animals

The protocol in this study was approved by the institutional review board of the First Affiliated Hospital of Nanchang University on the Use of Animals in Research and Teaching. All the methods in this study were performed in co‐ordination with relevant guidelines and regulations. Sprague Dawley (SD) rats were purchased from the animal science research department of the Jiangxi Traditional Chinese Medicine College (JZDWNO: 2011‐0030; Nanchang, Jiangxi,China). The learning and memory functions of the parental rats were assessed using the Morris water maze (MWM) system before mating, so that to minimize the hereditary difference. Animals were housed separately under standard laboratory conditions with 12:12 light/dark cycle, 24 ± 1°C and had free access to tap water. Two female rats in cages with one male rat per cage for mating. Pregnancy was diagnosed by the sign of vaginal plug.

### 2.3 Drug treatment

On E7, pregnant rats received intravenous infusion of propofol (n = 10 dams) with the rate of 20 mg kg^−1^ h^−1^ for 4 hours, equal volume of saline (n = 10 dams) or intralipid (n = 5 dams), respectively.

Electrocardiograms, saturation of pulse oximetry (SpO2) and tail non‐invasive blood pressure were continuously monitored during maternal propofol exposure. Using heating lamp and temperature controller to monitor the rectal temperature and keep it at 37 ± 0.5°C. Arterial blood sampling from lateral caudal artery for blood gas analysis at the end of propofol anaesthesia. If the total time of SpO2 <95% and/or the systolic blood pressure <80% of the baseline in excess of 5 minutes, the pregnant rat was got rid of the study, and other pregnant rats were chosen to supply the sample size, so as to exclude the interfering effect of maternal hypotension or hypoxia on cognitive function in the pup rats.

**Figure 1:**
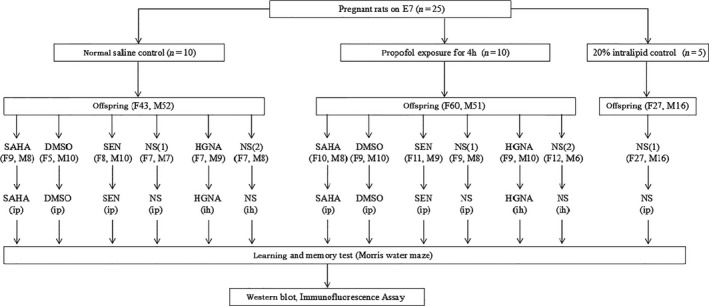
Experimental design. Pregnant dams were exposed to Propofol, 20% Intralipid or normal saline on E7, and the offspring were treated with SAHA, Senegenin, HGNA or vehicles two hours before behavioural testing. The number in parentheses represents the number of animals: F, female; M, male; SAHA, suberoylanilide hydroxamic acid, also known as vorinostat; DMSO, dimethyl sulphoxide; SEN, Senegenin; NS(1), Normal saline intraperitoneal injection; NS(2), Normal saline intrahippocampus injection; HGNA, HGN antisense oligonucleotide

After delivery, the offspring rats born to the same pregnant rat were randomly subdivided into the SAHA, SEN, HGNA group and their relative control groups (DMSO, NS(1) and NS(2) group, respectively; Figure 1). It has been proved that the acetylation level of histone in hippocampus obviously increased 2 hour after the administration of HDAC inhibitor.^27^ Therefore, 90 mg kg^−1^ SAHA (HDAC inhibitor), at a concentration of 0.6 μmol L^−1^ dissolved into dimethyl sulphoxide (DMSO) was injected to the offspring in SAHA group by the intraperitoneal route at 2 hours before each MWM trial. The same volume of DMSO was given to the DMSO group. Senegenin, a kind of Chinese medicine, was proved to up‐regulate the expression of NR2B mRNA and protein, thus to mitigate cognitive dysfunction.^28^ So, 15 mg kg^−1^ Senegenin and equal volume of saline were given intraperitoneally at 2 hours before each MWM trial to SEN or NS(1) groups, respectively. HGN antisense oligonucleotide (0.25 nmol μL^−1^, 1.5 μL) or normal saline (1.5 μL) was injected to offspring's hippocampus in HGNA or NS(2) group as previously described,^18^, ^29^ once daily for seven consecutive days before MWM trial.

### 2.4 Morris water maze test

Spatial learning and memory were assessed by the MWM test from post‐natal day 30 (P30) to P36 according to previously described^5^, ^30^ with SLY‐WMS Morris water maze test system (Beijing Sunny Instruments Co. Ltd., Beijing, China). Briefly, the trials start at 9 o'clock in the morning in the MWM system with the pool was filled with water to a height of 1.0 cm above the top of a 15‐cm‐diameter platform, in the second quadrant (target quadrant), and the water maintained at 24 ± 1°C. The training trial was performed once a day for six consecutive days. In each training trial, offspring rats were placed in the water facing the wall of the pool in the third quadrant, the farthest one from the target quadrant. The animals were allowed to search for the hidden platform or for 120 seconds. They were allowed to remain on the platform for 30 seconds when they found the platform and the time for the animal to find the platform was recorded as escape latency (indicating learning ability). For those who did not find the platform within 120 seconds, the animals were gently guided to the platform and allowed to stay there for 30 seconds, and their escape latency was recorded as 120 seconds. At the end of the reference training (P37), the platform was removed. The offspring rats were allowed to perform spatial probe test (memory function test) for 120 seconds. Times across the platform (platform crossing times, indicate memory function), the swimming trail and speed were automatically recorded by the system. The mean value of the platform crossing times, escape latency and speed of the offspring born to the same pregnant rats was taken as the final results.

### 2.5 Brain hippocampus harvest

The day after the MWM test, rats were anaesthetized with isoflurane and killed by cervical dislocation. Hippocampus tissues were harvested and stored in Eppendorf tubes that had been treated with 1% DEPC and were stored at −80°C (for Western blot analyses) or immersed in 4% paraformaldehyde (for immunofluorescence assay).

### 2.6 Western blot analysis

The hippocampus (n = 6, with three male and three female offspring rats from each group) were homogenized on ice in lysis buffer containing a protease inhibitors cocktail. Protein concentration was determined by the bicinchoninic acid protein assay kit. Protein samples (20 μg) were separated by sodium dodecyl sulphate polyacrylamide gel electrophoresis (SDS‐PAGE) and transferred to a PVDF membrane. The membranes were blocked by non‐fat dry milk buffer for 1.5 h and then incubated overnight at 4°C with antihistone H3 (acetyl K14) (1:2000), antihistone H4 (acetyl K12) (1:10000), anti‐NMDAR2B (1:1000), anti‐HGN (1:1000), antisynaptophysin (1:10000) and anti‐β‐actin (1:2000), respectively. Thereafter, the membranes were washed three times with TBS‐T buffer for 15 minutes and incubated with the horseradish peroxidase (HRP)‐conjugated secondary antibody for 2 hours at room temperature. The immune complexes were washed three times with TBS‐T buffer and detected using the ECL system (Millipore Corporation, MA, USA). The images of Western blot products were collected and analysed by ImageJ 1.50i (Wayne Rasband, National Institutes of Health, USA). The density of observed protein band was normalized to that of β‐actin in the same sample. The results of offspring from all the other group were then normalized to the average values of normal saline control offspring (control+NS group) in the same Western blot. The mean expression level of all of the offspring born to the same mother rat in the same group was calculated as the final expression level of the observed proteins.

### 2.7 Immunofluorescence staining

Immunofluorescence staining was used to assess HDAC2 and phospho‐CREB in the hippocampus of offspring rats after the MWM test. Hippocampus from offspring rats (n = 6, with three male and three female offspring rats from each group) were fixated in paraformaldehyde. Five‐μm frozen sections of the hippocampus were used for the immunofluorescence staining. The sections were incubated with anti‐HDAC2 (1:300) and anti‐CREB (1:100) dissolved in 1% bovine serum albumin in phosphate‐buffered saline at 4°C overnight. Then, the sections were incubated with fluorescent‐conjugated anti‐rabbit secondary antibody (1:300) for 1 hour in the dark at room temperature. Negative control sections were incubated with PBS as a substitute for primary antibody. Finally, the sections were wet mounted and viewed immediately using a inverted fluorescence microscope (200×) (Olympus, Japan). The target protein was red, and nuclei were blue. The proteins of HDAC2 and p‐CREB were excited by the green light, while the DAPI was performed by UV blue light. All images were recorded at 10 × 20× (Exp Acq‐700mmm, Offset Acq‐1, Gain Acq‐1, Gamma Acq‐300). The density of HDCA2 and p‐CREB staining was conducted on the images using Image‐Pro Plus 6.0 (Media Cybernetics Inc., USA). The images were converted it into black and white pictures. After intensity calibration, hippocampal CA1 area was chosen to analyse and the integrated optical density (IOD) was measured. IOD/Area was calculated as the protein expression level.

### 2.8 Statistical analysis

All analyses were performed with SPSS 17.0 software (SPSS, Inc., Chicago, IL, USA). Data from escape latency in the MWM test were subjected to a repeated measures two‐way analysis of variance (RM two‐way ANOVA) and were followed by least significant difference *t* (LSD‐*t*) analysis when a significant overall between‐subject factor was found (*P* < 0.05). Data from Western blot and immunofluorescence staining results were subjected to one‐way ANOVA analysis. All data well provided for any of the variables. The LSD *t* test was used to determine the difference between groups. Statistical significance was declared at *P* < .05.

## 3 RESULTS

### 3.1 Physiological parameters of maternal propofol anaesthesia

During propofol infusion, the maternal body temperature, respiratory rate, arterial oxygen saturation, heart rate and non‐invasive blood pressure were continuously monitored and recorded every five minutes. No significant change in these physiological parameters had been seen during propofol exposure (4 hours). Tail artery blood was collected from pregnant rats for blood gas analysis after propofol perfusion, and no significant difference (*P* > .05) was observed (Table 1). These results suggested that propofol has no side effect on the physiological parameters in pregnant rats, indicating the results of offspring rats in this study are likely caused directly by propofol rather than secondary effects of maternal propofol infusion.

**Table 1:** Maternal arterial blood gas at the end of propofol exposure or normal saline (n = 10, mean ± SD)

Indexes	Normal Saline exposure pregnant rats	Propofol exposure pregnant rats
pH	7.39 ± 0.04	7.38 ± 0.05
PO_2_ (mm Hg)	97.17 ± 3.49	94.00 ± 3.52
PCO_2_ (mm Hg)	45.33 ± 2.88	44.83 ± 5.78
${\text{HCO}}_{3}^{-}$ (mmol L^−1^)	27.95 ± 3.21	26.68 ± 2.32
K^+^ (mmol L^−1^)	3.47 ± 0.39	3.48 ± 0.29
Na^+^ (mmol L^−1^)	141.67 ± 1.03	140.83 ± 1.47
Ca^2+^ (mmol L^−1^)	1.38 ± 0.05	1.34 ± 0.03
Glu	9.18 ± 0.99	9.57 ± 0.55

### 3.2 Physical features of the offspring

**Figure 2:**
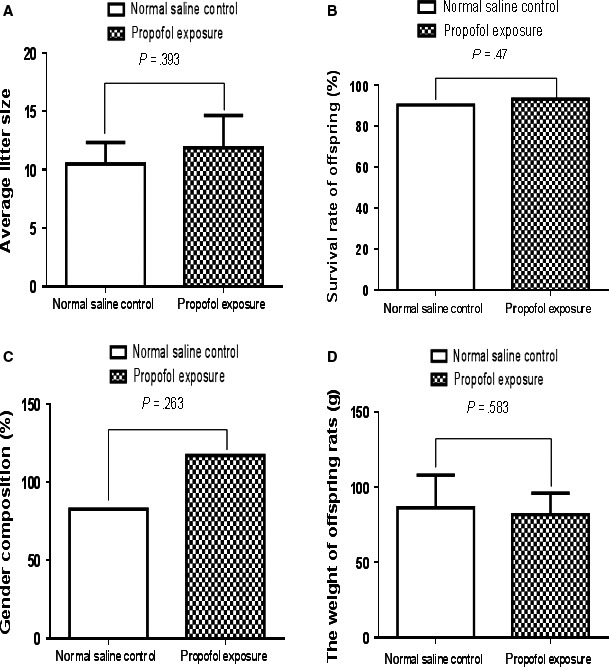
Maternal propofol exposure had no effect on the physical features of the offspring rats. The physical features of the offspring rats between propofol exposure and normal saline control group had no significant difference (*P* > .05). A, The birth rate (average litter size, total number of neonates born to each mother rat). B, Survival rate of offspring (survived more than 30 days). C, Gender ratio (the ratio of females to males, gender composition). D, The average weight of the offspring on day P30

The birth rate (total number of neonates born to each mother rat), survival rate (survived more than 30 days), gender ratio (the ratio of females to males) and the average weight of the offspring on day P30 in propofol exposure group were not significantly different from normal saline control group (Figure 2). Dyskinesia was not observed in either of the two groups. These results indicate that maternal propofol anaesthesia at the early pregnant stage (E7) has no significant effects on physical development of offspring rats, indicating the differences in learning and memory observed in this study are caused by propofol exposure during pregnancy rather than physical differences.

### 3.3 Impaired learning and memory in offspring and the ameliorating effect of SAHA, Senegenin and HGN antisense oligonucleotide

**Figure 3:**
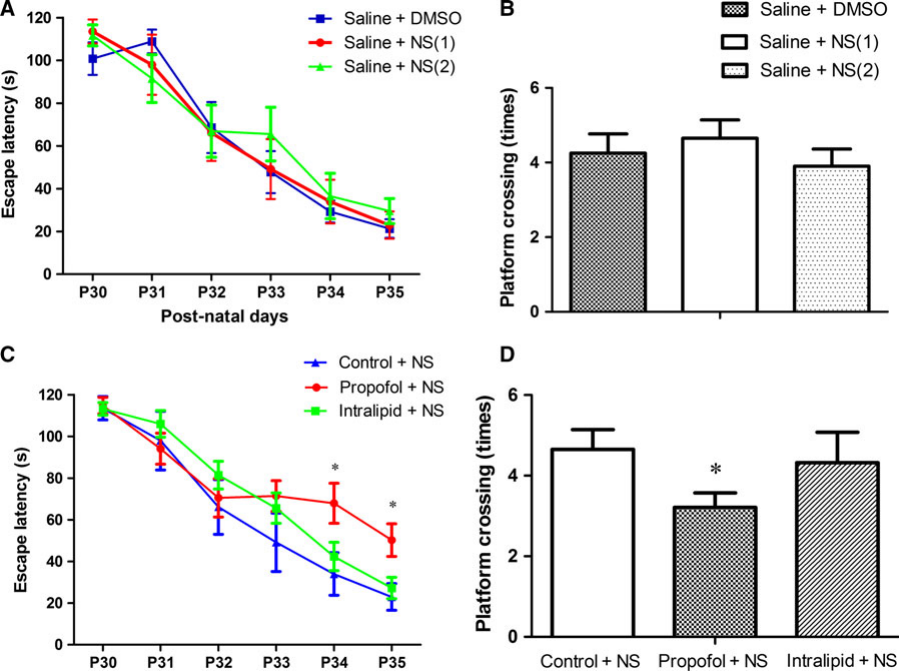
Maternal Propofol exposure impaired learning and memory in offspring. Post‐natal thirty days (P30), the learning and memory were assessed using the Morris water maze (mean ± SD). A, Escape latency (indicating learning ability) among control groups. B, Platform crossing times (indicating memory ability) among control groups. C, Propofol exposure increased escape latency in offspring compared to the saline control condition (*P < .05). No statistically significant difference was observed between the saline control and intralipid group. D, Propofol exposure decreased platform crossing times in offspring compared to the saline control condition (*P < .05). No significant difference was observed between the saline control and intralipid group

There was no obvious difference in offspring between normal saline and intralipid infusion group (Figure 3C,D). Therefore, we merged the data of offspring from normal saline and intralipid infusion group into one control group in the following data analysis. Propofol exposure increased escape latency, while decreased platform crossing times in offspring compared to the saline control condition (Figure 3C,D, P < .05), indicating propofol anaesthesia on E7 impairs spatial learning and memory in offspring.

**Figure 4:**
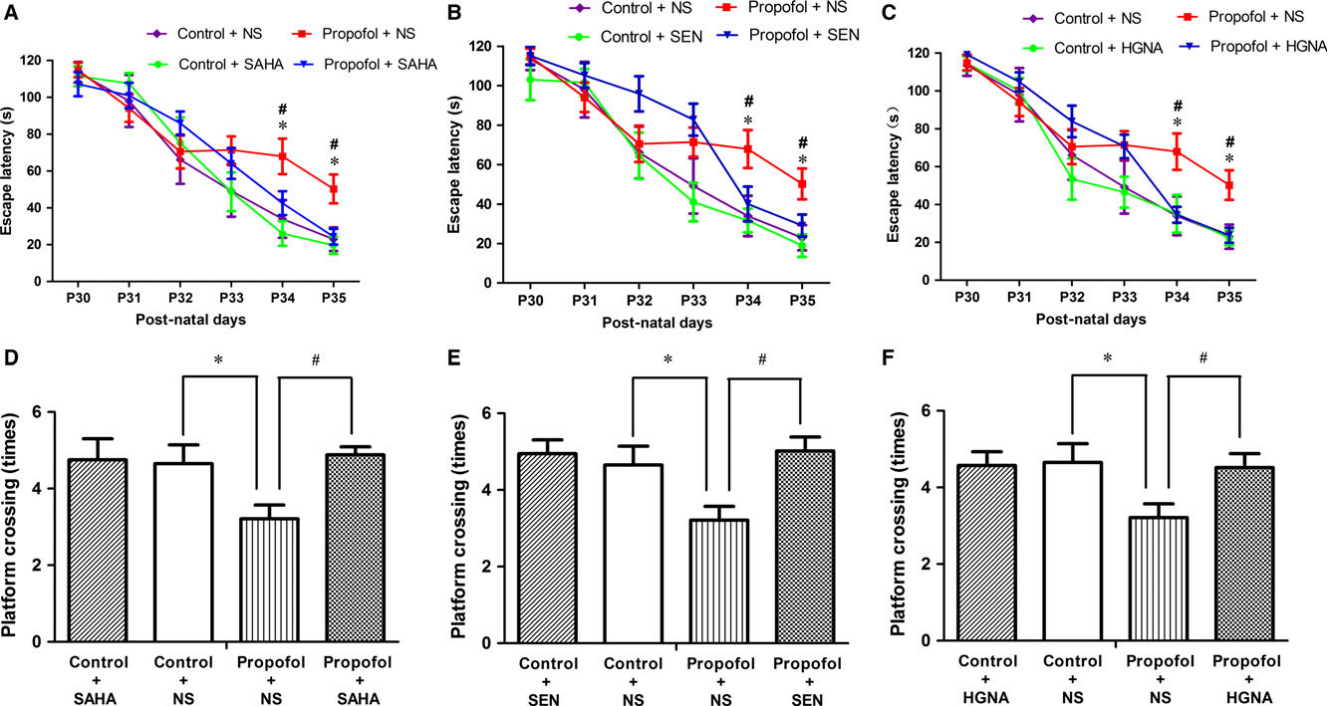
SAHA, SEN and HGNA treatment mitigated the learning and memory impairment (mean ± SD). A, Propofol exposure increased the escape latency in offspring compared to the control condition (*P < .05), and SAHA treatment significantly reversed the effect (^#^ *P* < .05). B, SEN treatment significantly reversed the effect (^#^ *P* < .05). C, HGNA treatment significantly reversed the effect (^#^ *P* < .05). D, Propofol exposure decreased the platform crossing times in offspring compared with the control condition (*P<.05), and SAHA treatment reversed the effect (^#^ *P* < .05). E, SEN treatment reversed the effect caused by propofol exposure (^#^ *P* < .05). F, HGNA treatment reversed the effect caused by propofol exposure (^#^ *P* < .05), and SAHA, SEN and HGNA treatment had no significant effect on learning and memory in offspring that were not exposed to propofol during pregnancy. Error bar = SD

SAHA, Senegenin and HGN antisense oligonucleotide have been shown to improve learning and memory by facilitating histone acetylation, increasing NR2B expression and inhibiting HGN expression, respectively.^18^, ^27^, ^28^ Therefore, we assessed whether they can ameliorate the learning and memory impairment caused by propofol exposure during pregnancy. Based on the previous discovery on the pharmacodynamics,^18^, ^27^, ^28^ SAHA or Senegenin was intraperitoneally injected into the offspring 2 hours before each MWM test, while HGN antisense oligonucleotide was injected into hippocampus 2 hours before each MWM test. The results showed that SAHA, Senegenin or HGN antisense oligonucleotide treatment ameliorated the cognitive function deficit caused by propofol exposure during pregnancy (Figure 4A‐F, P < .05). SAHA, Senegenin or HGN antisense oligonucleotide had no obvious effect on the learning and memory in offspring that had not exposed to propofol during pregnancy (Figure 4A‐F).

### 3.4 Reduced histone acetylation levels and the mitigating effect of SAHA,Senegenin and HGN antisense oligonucleotide

**Figure 5:**
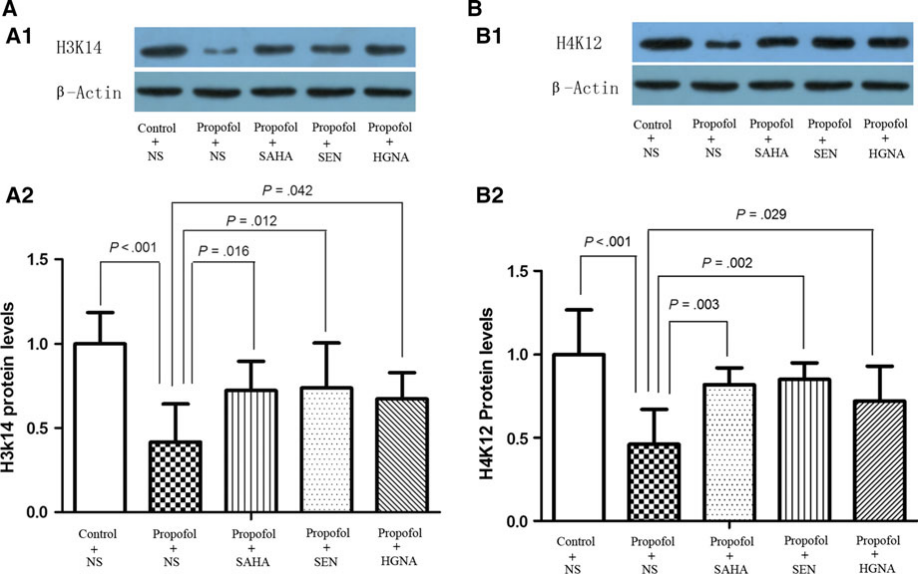
Maternal propofol exposure reduced the level of histone acetylation and the reversed effect of SAHA, SEN and HGNA treatment. Acetylation level of H3K14 and H4K12 was detected by Western blot (mean ± SD). Maternal exposure to propofol decreased the acetylation level of H3K14 and H4K12 in offspring compared to the control condition (*P* < .001), and SAHA treatment significantly increased acetylated H3K14 (*P* = .016) and H4K12 (*P* = .003) levels; SEN treatment significantly increased acetylated H3K14 (*P* = .012) and H4K12 (*P* = .002) levels; HGNA treatment significantly increased acetylated H3K14 (*P* = .042) and H4K12 (*P* = .029) levels. The protein levels of acetylated H3K14 and H4K12 in propofol + SAHA, propofol + SEN or propofol + HGNA group were not significantly different from those in control + NS group (*P* > .05)

**Figure 6:**
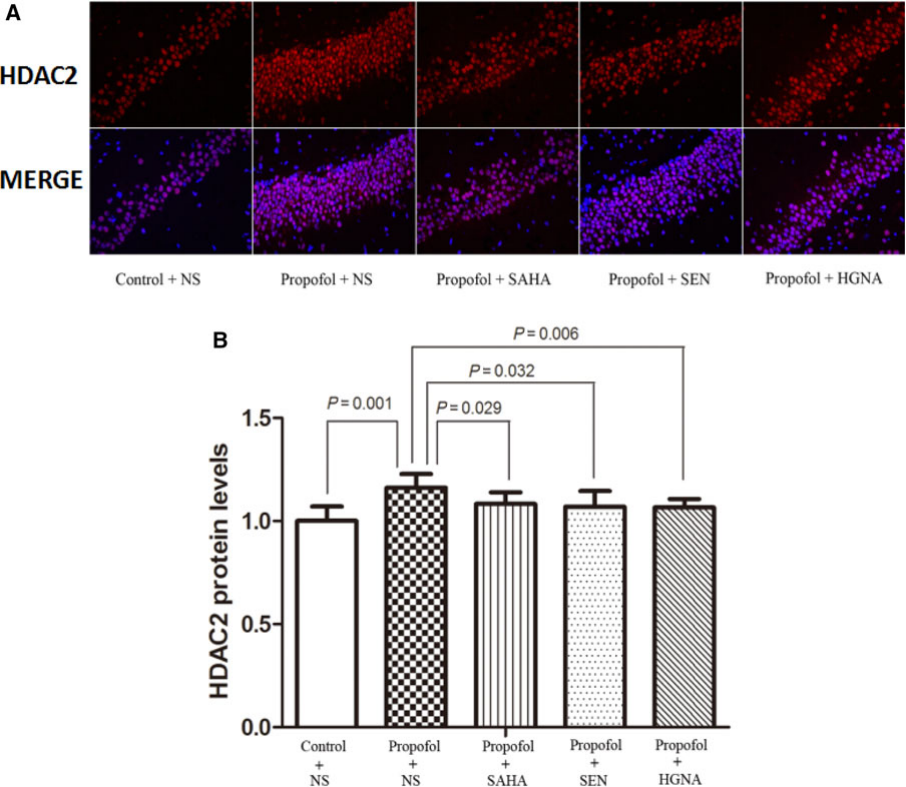
Maternal propofol exposure increased the level of HDAC2 and the reversed effect of SAHA, SEN and HGNA treatment. HDAC2 protein level was determined by immunofluorescence (mean ± SD). Maternal exposure to propofol up‐regulated the expression of HDAC2 protein in offspring compared to the control condition (*P* = .001). SAHA treatment significantly inhibited the expression of HDAC2 protein (*P* = .029); SEN treatment significantly decreased HDAC2 protein level (*P* = .032); HGNA treatment significantly decreased HDAC2 protein level (*P* = .006). The protein levels of acetylated HDAC2 in propofol+SAHA, propofol + SEN or propofol + HGNA group were not significantly different from those in control + NS group (*P* > .05)

Histone deacetylation was implicated in memory impairments.^31^, ^32^ The acetylation of histone is regulated by histone deacetylases (HDACs) and histone acetyltransferases (HATs).^33^, ^34^ HATs acetylate multiple lysine residues on histones, and different acetylated sites result in different downstream biological effects. H3K14 and H4K12 acetylation have been shown to play a crucial part in learning, memory and synaptic plasticity.^35^ The results showed that propofol exposure during pregnancy up‐regulated HDAC2 protein expression in offspring rat's hippocampus (Figure 6, P < .05), whereas decreased the acetylation levels of H3K14 and H4K12 significantly (Figure 5, P < .05). SAHA, Senegenin and HGN antisense oligonucleotide alleviated these changes (Figures 5 and 6, P < .05). These results indicate that propofol anaesthesia during pregnancy inhibits histone acetylation in offspring rats’ hippocampus, which could be alleviated by SAHA, Senegenin or HGN antisense oligonucleotide.

### 3.5 Decreased phosphorylated CREB levels in hippocampus and the mitigating effect of SAHA, Senegenin and HGN antisense oligonucleotide

**Figure 7:**
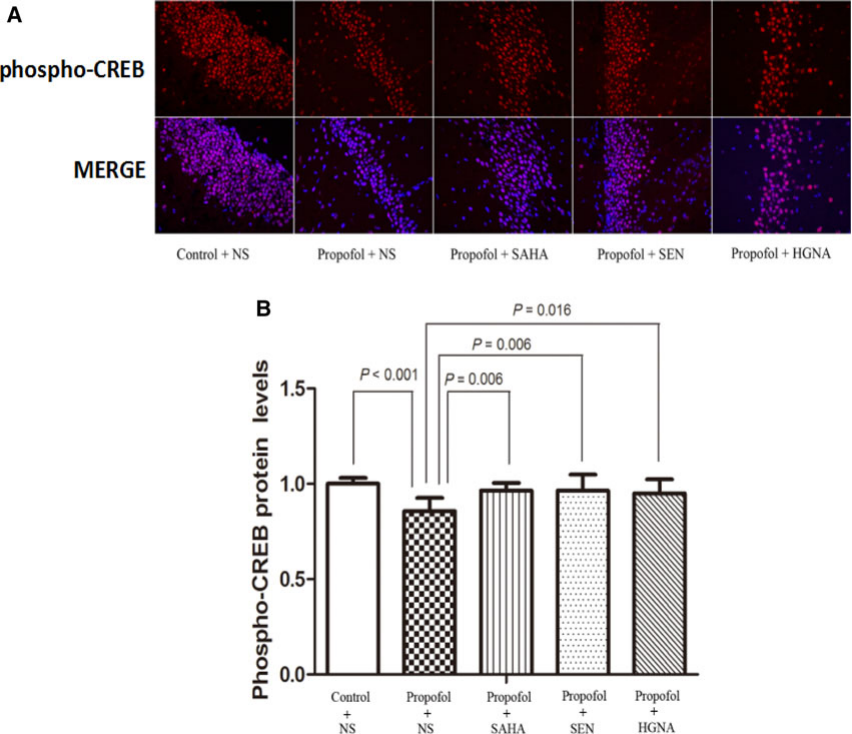
Maternal propofol exposure decreased the level of phospho‐CREB and the reversed effect of SAHA, SEN and HGNA treatment. Phospho‐CREB protein level was determined by immunofluorescence (mean ± SD). Maternal exposure to propofol decreased the expression of phospho‐CREB protein in offspring compared to the control condition (*P* < .001). SAHA, SEN and HGNA treatment significantly increased phospho‐CREB protein level (*P* = .006, *P* = .006, *P* = .016, respectively). The protein levels of phospho‐CREB in propofol + SAHA, propofol + SEN or propofol + HGNA group were not significantly different from those in control + NS group (*P* > .05)

Phosphorylation of CREB is recognized as a molecular marker of memory processing in the hippocampus for spatial learning.^36^ Therefore, we investigated the phosphorylation of CREB in this study. The results showed that propofol anaesthesia during pregnancy resulted in decrease in phospho‐CREB protein in offspring rats’ hippocampus. SAHA, Senegenin or HGN antisense oligonucleotide treatment alleviated the effects (Figure 7, P < .05). These results suggest that propofol anaesthesia during pregnancy on E7 can down‐regulate the phosphorylation of CREB in hippocampus of the offspring, whereas SAHA, Senegenin or HGN antisense oligonucleotide ameliorates this effect.

### 3.6 Decreased the ratio of NR2B/HGN in offspring rat's hippocampus and the reversing effect of SAHA, Senegenin or HGN antisense oligonucleotide

**Figure 8:**
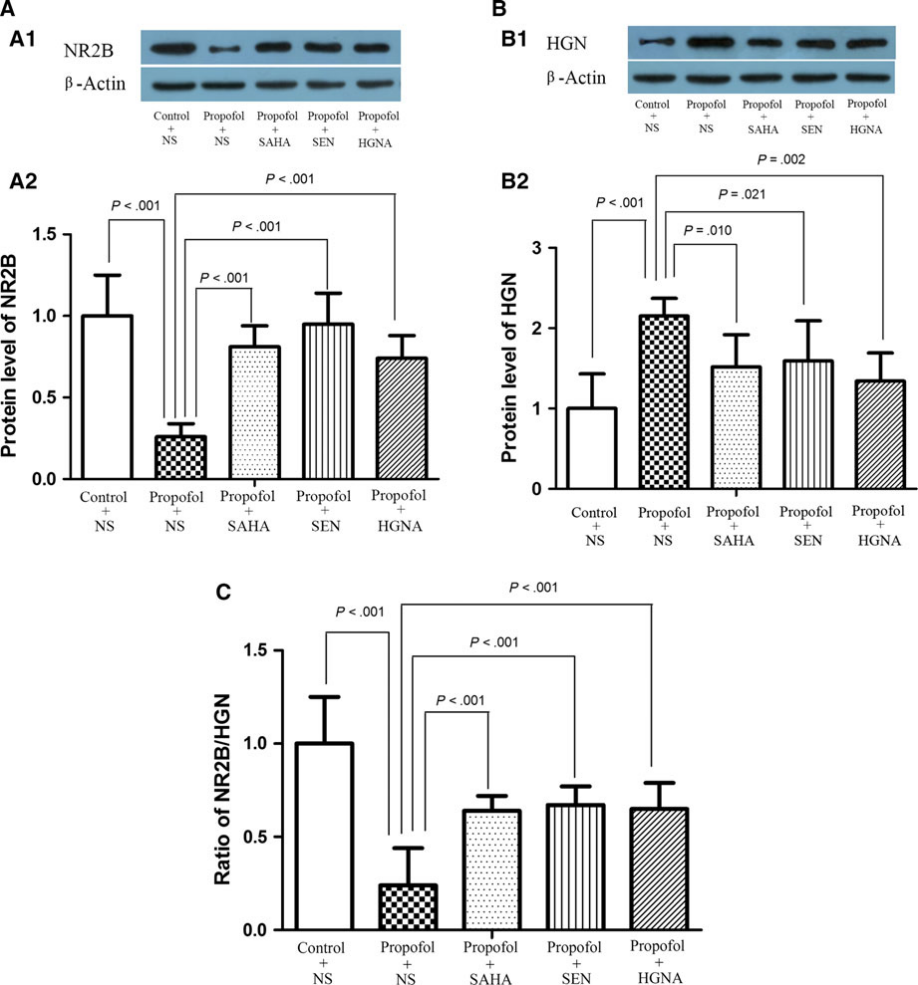
Maternal propofol exposure broken the balance between NR2B and HGN and the mitigating effect of SAHA, SEN and HGNA treatment. Expression of NR2B and HGN protein was detected by Western blot (mean ± SD). A, Maternal exposure to propofol decreased the NR2B protein level (*P* < .001). B, Maternal exposure to propofol increased the HGN protein level (*P* < .001). C, The ratio of NR2B/HGN was significantly reduced in offspring compared with the control condition (*P* < .001). SAHA, SEN and HGNA treatment significantly increased NR2B protein level and decreased HGN protein level and reversed the ratio of NR2B/HGN (*P* < .05). The protein levels of NR2B and HGN and the ratio of NR2B/HGN in propofol + SAHA, propofol + SEN or propofol + HGNA group were not significantly different from those in control + NS group (*P* > .05)

The balance between positive and negative regulating factors of learning and memory plays a key role in the memory obtain and maintenance.^37^ NR2B is recognized as critical positive regulating factor,^38^ while HGN is considered as an important negative factor.^18^ The results in this study showed that propofol anaesthesia during pregnancy resulted in decrease in NR2B protein (Figure 8A, P < .05), while increased the level of HGN protein (Figure 8B, P < .05), resulted in decreased ratio of NR2B/HGN in offspring rats’ hippocampus (Figure 8C, P < 0.05). The ratio of NR2B/HGN was reversed significantly by SAHA, Senegenin or HGN antisense oligonucleotide (Figure 8A‐C, P < .05).

### 3.7 Down‐regulated expression of synaptophysin in the hippocampus of offspring rats and the improving effect of SAHA, Senegenin and HGN antisense oligonucleotide

**Figure 9:**
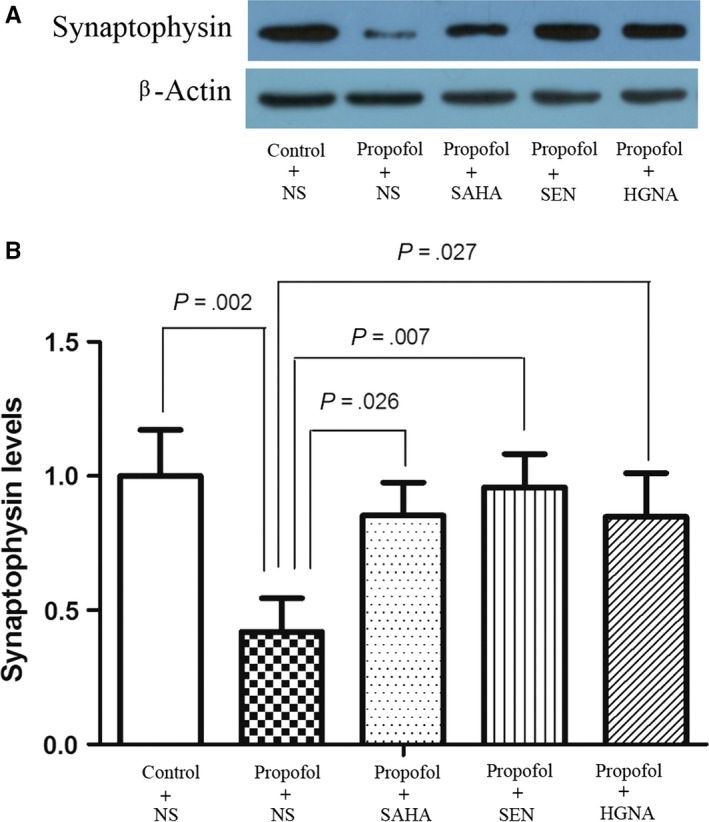
Maternal propofol exposure decreased the expression of synaptophysin and the reversed effect of SAHA, SEN and HGNA treatment. Synaptophysin level was determined by Western blot (mean ± SD). Maternal exposure to propofol decreased the expression of synaptophysin in offspring compared to the control condition (*P* = .002). SAHA, SEN and HGNA treatment significantly increased synaptophysin protein level (*P* = .026, *P* = .007, *P* = .027, respectively). The protein levels of synaptophysin in propofol + SAHA, propofol + SEN or propofol + HGNA group were not significantly different from those in control + NS group (*P* > .05)

Synaptophysin plays an important role in the exocytosis of synaptic vesicles and acknowledged as a marker of synaptic density.^39^ Synapse loss is closely associated with cognitive dysfunction and learning impairment.^40^, ^41^ The results showed that the protein level of synaptophysin in maternal propofol exposure group was lower than control condition (Figure 9), indicating maternal propofol exposure on E7 impairs the synaptic plasticity in offspring rats’ hippocampus, whereas the level of synaptophysin in SAHA, Senegenin or HGN antisense oligonucleotide‐treated group was higher than propofol exposure group (Figure 9), suggesting that SAHA, Senegenin and HGN antisense oligonucleotide can reverse the down‐regulated expression of synaptophysin caused by propofol.

## 4 DISCUSSION

The current study findings suggest that pregnant rats propofol anaesthesia on E7 impairs the learning and memory in offspring rats, increases the expression of HDAC2, inhibits the acetylation of H3K14 and H4K12 and the phosphorylation of CREB, down‐regulates the expression of NR2B, up‐regulates the expression of HGN and decreases the ration of NR2B/HGN and the expression of synaptophysin. SAHA, Senegenin and HGN antisense oligonucleotide ameliorate all these changes.

Maternal body temperature, respiratory rate, saturation of pulse oximetry, heart rate and non‐invasive blood pressure were continuously monitored during propofol exposure, and no obvious abnormality was observed. Furthermore, maternal artery blood gases were analysed after the 4 hours propofol infusion and showed no significant change (Table 1). Moreover, there was no significant difference in birth rate, offspring survival rate, the ratio of sex or basic physical development of offspring between propofol and saline group. These results suggested that the impaired learning and memory of the rats’ offspring may be not caused by pathological disorders but caused by the pregnant rats propofol anaesthesia in the current study.

Several animal studies showed that anaesthetics exposure during gestation induced apoptosis in foetal brain.^1^, ^2^, ^40^ Xiong et al and our previous study showed that prenatal propofol exposure resulted in learning and memory deficit in offspring.^4^, ^5^ While these studies mainly focused on the second and third trimester, there is little information in relation to the effect of propofol anaesthesia during early pregnancy on the cognitive function in offspring. Because some of non‐obstetric surgeries during pregnancy occurred in the first trimester,^10^ our current study mainly focus on gestation day 7, which distinct with the exposure time‐point in previous studies,^4^, ^5^, ^6^ the different exposure time‐point may alter the vulnerability to general anaesthetics for the developing brain. Halothane and enflurane exposure on gestation day 6 and 10 (amount to the early and early the 2nd trimester of pregnancy) may cause learning deficits in the rat offspring.^42^ Our previous study showed that ketamine, propofol and enflurane anaesthesia during early gestation (on gestation day 7) induced learning and memory impairment in offspring rats,^11^, ^12^, ^13^, ^14^ associated with hippocampal neuron injury, NR2B receptor subunit reduction and increased level of HGN mRNA.

How does propofol anaesthesia during pregnancy impair the learning and memory in offspring? The consolidation and maintenance of memory require specific genes expression, and histone acetylation promotes the expression of these genes, while histone deacetylation represses their expression.^21^, ^43^ Histone deacetylases (HDACs) inhibit the expression of these genes, while histone acetyltransferases (HATs) promote their expression.^44^ Among the HDACs, HDAC2 was implicated in learning and memory, it negatively regulates synaptic plasticity and memory process by suppressing memory specific genes’ expression, and loss function of HDAC2 facilitates synaptic plasticity and learning and memory.^32^ Graff et al showed that HDAC2 overexpression reduced the histone acetylation of histone and inhibited the expression of memory specific genes. HDAC2 is significantly enriched near the histones of genes shown to play a key role in learning, memory and synaptic plasticity, such as H2B lysine (K) 5, H3K14, H4K5, and H4K12. Reversing the build‐ up of HDAC2 by short‐hairpin‐RNA ‐mediated knockdown activated these genes, reinstated structural and synaptic plasticity and abolished the neurodegeneration‐associated memory impairments. Abolished the memory impairments in connection with neurodegeneration.^35^ Our earlier study suggested that maternal isoflurane anaesthesia during third trimester impairs the spatial learning and memory of the offspring rats, and its mechanism in connection with the up‐regulation of HDAC2 mRNA and subsequent inhibits the expression of CREB mRNA and NR2B, while HDAC2 inhibition reversed these changes.^30^ Consistent to our previous study, our results suggest that maternal propofol anaesthesia on E7 impairs learning and memory in offspring rats, causes the overexpression of HDAC2 and inhibits the acetylation of H3K14 and H4K12, and these effects were reversed by SAHA. Senegenin and HGN antisense oligonucleotide treatment also showed similar effects.

NMDA receptors play a crucial role in neuronal development and circuit formation. Subunit NR2B is critical to learning and memory.^45^ It is reported that the enhancement of pre‐frontal cortical long‐term potentiation (LTP) and working memory *via* the up‐regulate expression of NR2B specifically in the forebrain region.^46^ While decreased expression of NR2B subunit suppressed NMDA‐dependent long‐term potentiation (LTP) and impaired spatial learning.^47^ Therefore, NR2B acts as a positive regulator in memory process by promoting synaptic plasticity and long‐term potential (LTP). Not only the activation of positive regulatory mechanisms that favour memory storage but also the removal of inhibitory constraints that prevent memory storage are required for long‐lasting of synaptic plasticity.^37^ Negative regulators play an important role in the formation and maintenance of memory. Hippyragranin (HGN) is a protein which expresses in rat hippocampus and involves in negative memory regulation.^18^ Down‐regulation of HGN by antisense oligonucleotide in the hippocampal CA1 region caused enhanced learning and memory as well as elevated LTP. Therefore, we hypothesize that the balance between the positive regulator NR2B and negative regulator HGN plays a pivotal role in the learning and memory process. Present study showed that Senegenin treatment reversed the protein levels of NR2B, HGN and the ratio of NR2B/HGN, as well as enhanced learning and memory, which in accordance with the previous research that Senegenin attenuates cognitive impairment by up‐regulating expression of hippocampal NR2B expression in rats.^28^ While treatment with HGN antisense oligonucleotide inhibited the expression of HGN protein, reversed the ratio of NR2B/HGN and the learning and memory impairment as previous report.^18^

Transcription factor CREB (cAMP response element‐binding protein) shows an important role in synaptic plasticity underlying learning memory.^48^, ^49^ CREB is a critical mediator of cAMP‐ and calcium‐inducible transcription, whereas the phosphorylation of serine 133 (phospho‐Ser133) in its kinase‐inducible domain (KID) is its main transactivating form. Phospho‐Ser133 plays a role in CREB to bind the KIX domain of the coactivators CBP and p300 (CBP/p300).^50^ Vecsey et al^25^ demonstrated that enhancement of hippocampus‐dependent memory and synaptic plasticity by HDAC inhibitors was relied on the binding of CREB and CREB‐binding protein (CBP), which induced robust activation of gene transcription afterwards. The activity of CREB is essential to the gene transcription of NR2B, and expression of NR2B relies on the binding of p‐CREB to its binding site at the promoter of the NR2B gene.^51^ Fujita et al^27^ have demonstrated that HDAC inhibitor up‐regulated the expression of acetylated histones and NR2B mRNA in the hippocampus, and up‐regulated expression of acetylated histones was accompanied by enhanced binding of p‐CREB to its binding site at the promoter of the NR2B gene.^27^ These findings indicated that HDAC inhibitor promotes learning and memory by increasing the acetylation of histone and the phosphorylation of CREB, and subsequent increase of NR2B expression. Our previous study has demonstrated that isoflurane anaesthesia during the third trimester impaired learning and memory in offspring rats *via* “HDAC2‐CREB‐NR2B” pathway.^30^

Synaptophysin is a synaptic protein marker and provides a structural basis for synaptic plasticity.^52^ Decrease in synaptophysin is implicated in learning and memory impairment.^1^, ^4^, ^5^ Graff et al^35^ demonstrated that HDAC2 overexpression reduced synaptophysin protein level and caused memory impairments, HDAC2 inhibition reversed the effects. As synaptophysin is one of the CREB target genes,^53^ we detected the expression of synaptophysin in the present study. The results showed that propofol anaesthesia during pregnancy reduced the protein level of synaptophysin in offspring's hippocampus, whereas SAHA, Senegenin and HGN antisense oligonucleotide mitigated the reduced synaptophysin levels; meanwhile, the increased expression of synaptophysin was companied with decreased HDAC2 protein level, increased histone acetylation and CREB phosphorylation level. The BDNF‐TrkB signalling pathway is one of the downstream regulating targets of histone acetylation, so BDNF‐TrkB signalling pathway may be one of the underlying downstream mechanisms of learning and memory deficits induced by propofol exposure during early gestation. It is confirmed that HDAC2 up‐regulation will impair BDNF‐TrkB signalling pathway and results in cognitive impairments induced by isoflurane.^54^ Our previous study also verified the role of BDNF‐TrkB signalling pathway in the cognitive deficits induced by propofol during late pregnant stage.^5^ Whether BDNF‐TrkB signalling pathway involves in the learning and memory impairments induced by maternal propofol anaesthesia needs to be explored in future study.

Present study has several limitations. First, we had not accessed the pathological changes of neurons in the foetal brains immediately after maternal propofol exposure and during various period of brain development (e.g., post‐natal day 1 to 10). Second, in the present study, we only used MWM to evaluate learning and memory. Although MWM is recognized as an appropriate way to evaluate the spatial learning and memory in rodents, to provide a more comprehensive assessment of learning and memory in rat offspring, multiple behavioural test such as open field test, step‐through test and the fear conditioning test should be used in future study. Third, we have explored the underlying mechanisms only from hippocampus. Maternal propofol exposure may also affect other brain regions, such as cortex, thalamus and hypothalamus regions. Li et al^55^ found that propofol anaesthesia in pregnant rats induced caspase‐3 activation and microglial response in foetal rats. They found that the activated caspase‐3‐positive cells were abundant and heavily concentrated in the cortex, thalamus and hypothalamus regions.^55^ Whether maternal propofol anaesthesia will affect the histone acetylation in other brain regions should be studied. We had only evaluated the short‐term therapeutic effects of SAHA, Senegenin and HGNA on behaviour performance and proteins. The long‐term or long‐lasting therapeutic effects of these drugs on learning and memory deficits and protein expression changes caused by propofol exposure on E7 should be evaluated in future study.

Taken together, the results of the present study suggest that propofol anaesthesia during first trimester causes learning and memory deficit in offspring rats by inhibiting histone acetylation. SAHA, Senegenin and HGN antisense oligonucleotide can ameliorate these impairments.

## CONFLICT OF INTERESTS

The authors declare that they have no conflict of interest.
